# High Body Fat as a Predictor of Osteoporosis Risk in Postmenopausal Women: Insights From a Community-Based Cross-Sectional Study in Rural South India

**DOI:** 10.7759/cureus.59239

**Published:** 2024-04-28

**Authors:** Sahanaa Yuvaraja, Roy A Daniel, Yuvaraja Murugan, Vijayalakshmi Sridharan, Kokilaa G Latha, Kavya Palanisamy, Rathibala Arumugaperumal, Vinoth Thanikachalam

**Affiliations:** 1 Community Medicine, Chettinad Hospital & Research Institute, Chennai, IND; 2 Orthopaedics, Chettinad Hospital & Research Institute, Chennai, IND

**Keywords:** waist hip ratio, bmi-for-age, osteoporosis self-assessment tool for asians (osta), community-based study, rural, high body fat, postmenopausal women, osteoporosis

## Abstract

Introduction: Osteoporosis poses a significant health burden, particularly among postmenopausal women. While obesity in the form of BMI has been implicated in various health conditions, the relationship between waist-hip ratio (WHR) and osteoporosis remains debated. This study aims to estimate the prevalence of osteoporosis risk and explore the association between WHR and osteoporosis risk among postmenopausal women in rural South India.

Methods: A community-based cross-sectional study was conducted in the Chengalpattu district of Tamil Nadu. The study enrolled 435 postmenopausal women aged 45 years and above and the data were collected on socio-demographic characteristics, anthropometric measurements, and osteoporosis risk assessment using the Osteoporosis Self-assessment Tool for Asian Women (OSTA) scale. Logistic regression analysis was performed to identify factors associated with osteoporosis risk with 95%CI.

Results: The mean (SD) age of participants was 54.5 (8.6) years, 87% were married, 33% were illiterate with mean (SD) WHR of 0.88 (0.1). Around 80.5% of the participants were categorized as low risk, 16.1% as intermediate risk, and 3.5% as high risk based on OSTA scores. Older age, lower educational attainment, and higher waist-hip ratio were significantly associated with increased osteoporosis risk.

Conclusion: This community-based study found a 20% osteoporosis risk among postmenopausal women using the OSTA scale, with age, lower education, and waist-hip ratio as key determinants. Early identification and interventions, particularly targeting older and obese individuals, are crucial to alleviate the burden and complications of osteoporosis.

## Introduction

Osteoporosis, characterized by decreased bone mineral density and structural deterioration of bone tissue, is a pervasive and debilitating condition affecting millions of individuals worldwide, particularly postmenopausal women [1]. Osteoporosis can be classified into primary (postmenopausal and senile) and secondary forms, with primary types occurring without a specific underlying cause and secondary types associated with identifiable factors like malabsorption, medications (e.g., glucocorticoids), and certain diseases (e.g., hyperparathyroidism) [2]. Global deaths and disability-adjusted life years (DALYs) attributable to low bone mineral density (LBMD) increased substantially from 1990 to 2019. DALYs related to LBMD increased by 93.82%, and LBMD-related fracture DALYs rose by 121.07% during this period [3]. Osteoporosis and its associated fractures not only profoundly affect individuals' health and well-being but also impose a substantial financial burden on healthcare systems [4].

In 2019, India faced the highest burden of fractures related to LBMD in terms of DALYs, adding significantly to the global impact. The prevalence of osteoporosis in India is on the rise ranging from 8% to 62% [5], reflecting similar trends seen in other developing countries due to an aging population. Recent studies show an increasing number of fractures related to osteoporosis, especially among older adults, which presents significant challenges for healthcare systems and society as a whole [3]. Various risk factors contribute to osteoporosis development and progression, including age, gender, hormonal changes (such as menopause), genetic predisposition, sedentary lifestyle, smoking, excessive alcohol consumption, and inadequate dietary intake of calcium and vitamin D [6]. Complications associated with osteoporosis extend beyond fractures, encompassing substantial morbidity and mortality, diminished quality of life, functional impairment, and increased healthcare costs. Hip fractures, in particular, are linked to a heightened risk of disability, institutionalization, and premature death, underscoring the urgent need for preventive strategies and effective management approaches [7].

Despite the growing recognition of osteoporosis as a major public health concern, community-based screening initiatives for early detection and prevention remain limited, particularly in resource-constrained settings. The lack of accessible and affordable screening tools contributes to underdiagnosis and undertreatment of osteoporosis, exacerbating the burden of fractures and associated morbidity. Moreover, the unique socio-cultural and environmental factors in rural South India necessitate tailored approaches to healthcare delivery and preventive interventions [8]. The gold standard for diagnosing osteoporosis is dual-energy X-ray absorptiometry (DEXA) scan, and this is not widely available across India. In community-based settings, the need for efficient and cost-effective screening tools for osteoporosis is paramount. The Osteoporosis Self-Assessment Tool for Asians (OSTA), developed specifically for Asian populations, offers a practical and accessible approach to identify individuals at increased risk of osteoporosis. The OSTA tool utilizes age and body weight to estimate the likelihood of low bone mass, providing a convenient screening method that does not require specialized equipment or extensive training [9]. Moreover, the validation studies conducted in diverse populations have demonstrated the reliability and accuracy of the OSTA tool in predicting osteoporosis risk, making it a valuable tool for community-based osteoporosis screening initiatives [10].

Hence this study was conducted, 1. to estimate the prevalence of osteoporosis risk among postmenopausal women in a rural community using the OSTA scale and 2. to assess the association between body fat, as indicated by waist-hip ratio (WHR), and the risk of osteoporosis among these women. The findings of this study hold significant implications for public health practice, policy development, and clinical management of osteoporosis and can inform tailored interventions to mitigate the burden of osteoporosis-related fractures and improve bone health outcomes among postmenopausal women.

## Materials and methods

This community-based cross-sectional study was conducted in the rural areas of Chengalpattu district, Tamil Nadu. The study area comprises eight villages and one village was randomly selected for inclusion. The sampling frame included all women aged 45 years and above from the selected village. Sample size calculation was based on a 52% prevalence obtained from a similar study [11] using the OSTA tool, with 5% absolute error, 15% non-response rate, and 95% confidence interval, resulting in a required sample size of 440. Using random selection without replacement, 440 women were chosen from the sampling frame by a trained statistician. Data collection took place over two months (January and February 2024) through house-to-house visits conducted by trained data collectors. Efforts were made to visit all households, with a maximum of two attempts made for locked houses before excluding them. All the women aged 45 years and older who were residents of that village for at least six months were included in the study. We excluded those participants who were chronically ill and unable to comprehend the questionnaire from the study. Prior to participation, each potential participant was provided with a Participant Informed Consent Form (PICF) and Participant Information Sheet (PIS). Informed consent was obtained from each participant before conducting the interview schedule.

Trained MBBS students served as data collectors after undergoing a two-day training session covering study objectives, tools, and methodology. The study utilized a pre-tested semi-structured interview schedule, comprising socio-demographic details, anthropometric measurements (height, weight, waist circumference, and hip circumference in centimetres), and administration of the OSTA scale. Socio-demographic information collected included name, age, address, contact number, family size, monthly income, number of children, education, employment, history of chronic illnesses (e.g., hypertension, diabetes), medication history (e.g., oral contraceptives, glucocorticoids), and past fracture history. Anthropometric measurements adhered to WHO STEPS recommendations [12], with weight measured to the nearest 0.1 kg using an electronic scale and height measured to the nearest 0.5 cm with a portable stadiometer. Waist and hip circumferences were measured using a tape measure in centimetres. Standardization of equipment was ensured weekly using a standard weight for the scale and a portable stadiometer for the tape measure, with records maintained in a logbook.

OSTA is an index for classifying the risk of osteoporosis among Asians. Among the various scales used for identifying the at-risk population for osteoporosis, OSTA has the maximum sensitivity and specificity. This tool is validated in India and has been used widely for various epidemiological surveys. This tool uses two parameters: age of the women (in years) and weight (in kg). The index is calculated utilising the formula of 0.2 × [body weight (in kg) − age (years)] [13]. Participants were classified into three categories according to their age group. We defined “at-risk” as women who either had an intermediate or high-risk index. The index comprises three categories: (i) Low Risk Index > −1 (ii) Intermediate Risk Index −1 to −4 and (iii) High Risk Index < −4 [10]. The sensitivity of the OSTA scale with a cut-off of -1 is 91% and the Area Under the Curve (AUC) is 0.79, which is higher than other scales used to identify people at risk for osteoporosis [14]. The questionnaire was translated into Tamil (local vernacular language) by a trained translator and back-translated to check for consistency. The study protocol was approved by the Institute Ethics Committee number CARE IHEC-II/0481/24.

Statistical analysis

The data were entered into Microsoft Excel (Redmond, WA, USA) and analyzed using STATA version 16 (StataCorp., College Station, TX, USA). Categorical variables were summarized as frequencies and percentages. The normality of quantitative variables was assessed through histograms and the Shapiro-Wilk test. Quantitative variables were reported as Mean (SD) or Median (interquartile range (IQR)) based on their distribution. The prevalence of osteoporosis risk was estimated with a 95% Confidence Interval (CI). Linear regression analysis was performed with the outcome variable (Risk/No Risk of osteoporosis) and other independent variables. Variables with a p-value below 0.20 were included in the multivariable model to obtain adjusted B coefficient, and a p-value less than 0.05 was considered statistically significant. Multicollinearity was tested and variables with Variance Inflation Factor (VIF) of more than 5 were removed from the multivariable linear regression analysis.

## Results

Out of the 440 women approached, 435 participated in the study, yielding a response rate of 98.8%, indicating the adequacy of the sample size for estimating osteoporosis risk among postmenopausal women. The mean (SD) age of participants was 54.5 (8.6) years, with the majority of 60% falling within the 45 to 55 years age group. Nearly 87% of the women were married, while 10.6% were widows. Regarding education, 33.8% were illiterate, 52.5% had primary to high school education, and 13.8% possessed a degree. Over 71% were homemakers and had two children. The median monthly family income was 14,000 Indian Rupees (INR) (IQR 10,000-20,000) as shown in Table 1.

**Table 1:** Distribution of study participants based on socio-demographic variables N=435 IQR: interquartile range, INR: Indian Rupee

S. No	Socio-demographic variables	n (%)
1	Age group (in years)	
	45-55	265 (60.9)
	Above 55	170 (39.1)
	Mean (SD): 54.5 (8.6)	
2.	Marital status	
	Married	380 (87.4)
	Unmarried	6 (1.38)
	Widow	46 (10.6)
	Separated	3 (0.7)
3.	Educational status	
	Illiterate	147 (33.8)
	Primary School	70 (16.1)
	Middle school	88 (18.9)
	Senior school	32 (7.4)
	High School	44 (10.1)
	Diploma & graduates	60 (13.8)
4.	Employment	
	Employed	123 (28.3)
	Homemaker	312 (71.7)
5.	Number of children	
	Up to 2	312 (71.7)
	More than 2	123 (28.3)
	Median (IQR): 2 (1,3)	
6.	Monthly family income (INR) Median (IQR)	14,000 (10,000-20,000)

Regarding the clinical history, 46% reported comorbidities like diabetes, hypertension, or heart disease. A majority (93.6%) had no history of communicable diseases, such as coronavirus disease 2019 (COVID-19) or tuberculosis. Approximately 2% had a history of hormonal therapy usage. Nine percent of the study participants had a prior history of fractures. The mean (SD) age at menarche and menopause was 14.2 (1.5) and 47.9 (4.0) years respectively, with 61.6% having reached menopause. The mean (SD) weight was 62 (9.1) kg, height was 154.4 (5.5) cm, and waist-hip ratio was 0.88 (0.1) as shown in Table 2.

**Table 2:** The distribution of study participants based on anthropometric measurements N=435

S. No	Anthropometric measurements	Mean (SD)
1	Weight (in kg)	62. (9.1)
2	Height (in cm)	154.4 (5.5)
3	Waist circumference (in cm)	90.1 (9.9)
4	Hip circumference (in cm)	101.3 (12.1)
5	Waist hip ratio	0.88 (0.1)

**Figure 1:**
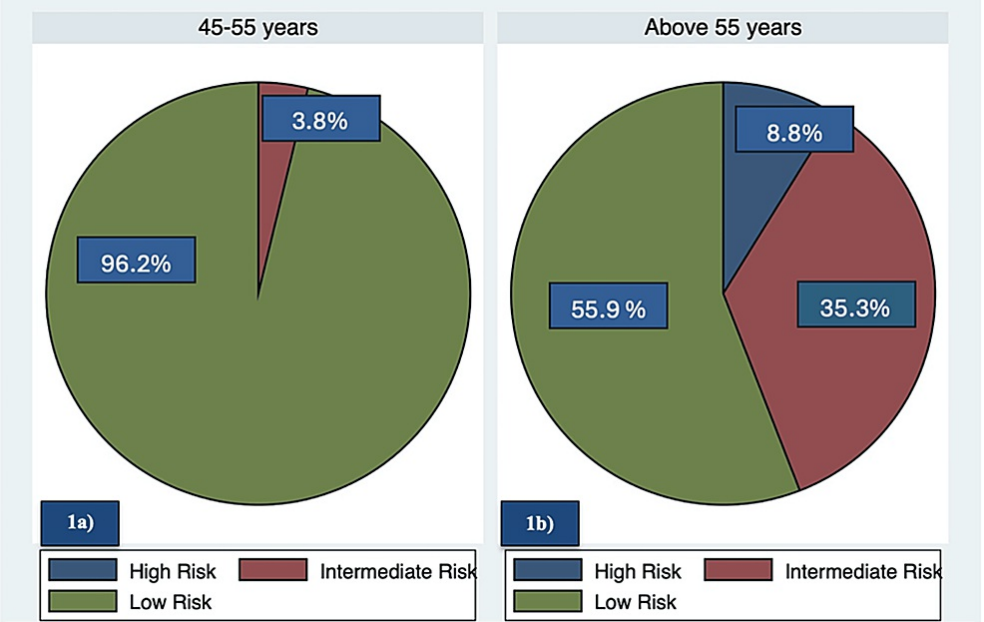
Prevalence of osteoporosis risk based on age group N=435. a) shows the prevalence of osteoporosis risk of postmenopausal women between 45 to 55 years. b) shows the prevalence of osteoporosis risk of postmenopausal women above 55 years.

Using the OSTA risk calculation formula, 80.5% (95%CI: 76.4-84.1) were at low risk, 16.1% (95%CI: 12.8-19.9) at intermediate risk, and 3.5% (95%CI: 1.9-5.6) at high risk. The risk is more pronounced after the age of 55 years as shown in Figure 1A, 1B. For regression analysis, the intermediate and high-risk groups were combined into a single risk group, while the low-risk group was considered a no-risk group.

In bivariable linear regression, age [unadjusted B coefficient UC -0.22 (95%CI: -0.24, -0.20)], having more than two children [UC -1.27 (95%CI: -1.86, -0.69)], schooling [UC 2.50 (95%CI:1.90, 2.91)], diploma [UC 2.68 (95%CI: 1.89, 3.47)], homemaker [UC -0.58 (95%CI: -1.17, 0.02)], age at menarche [US 0.18 (95%CI: -0.01, 0.36)], attained menopause [UC -2.37 (95%CI: -2.88, -1.86)] and waist-hip ratio [UC -6.16 (95%CI: -9.9, -2.3)] came as statistically significant. The variables years since menopause had a VIF value of more than 5 and hence were removed from the multivariable regression model. However, in multivariable linear regression, age [adjusted B coefficient AC -0.20 (95%CI: -0.22, -0.17)], schooling [AC 0.95 (95%CI: 0.48, 1.42)], diploma [AC 0.79 (95%CI: 0.13, 1.46)], and waist-hip ratio [AC -5.78 (95%CI: -8.55, -3.01)] came as statistically significant as shown in Table 3.

**Table 3:** Bivariable and multivariable linear regression to find the factors associated with OSTA score (N=435)* *The adjusted R2 for the model is 48.8% and the value of root mean squared error (MSE) is 2.04. OSTA: Osteoporosis Self-assessment Tool for Asian Women

Variable	Mean (SD) OSTA score	Bivariable model		Multivariable model	
		Unadjusted B coefficient (95%CI)	P -value	Adjusted B coefficient (95%CI)	p-value
Age	1.5 (2.8)	-0.22 (-0.24, -0.20)	<0.001	-0.20 (-0.22, -0.17)	<0.001
Marital status					
Unmarried	0.5 (3.2)	Reference			
Married	1.6 (2.7)	1.15 (-1.15, 3.44)	0.33		
Widow	0.4 (3.3)	-0.18 (-2.60, 2.24)	0.89		
Separated	0.7 (2.1)	0.13 (-3.80, 4.08)	0.95		
No. of children					
Less than 2	1.9 (2.7)	Reference			
More than 2	0.6 (2.9)	-1.27 (-1.86, -0.69)	<0.001	-0.30 (-0.75, 0.14)	0.18
Educational status					
Illiterate	-0.14 (2.8)	Reference			
Schooling	2.3 (2.4)	2.50 (1.90, 2.91)	<0.001	0.95 (0.48, 1.42)	<0.001
Diploma	2.6 (2.3)	2.68 (1.89, 3.47)	<0.001	0.79 (0.13, 1.46)	0.02
Employment status					
Employed	1.9 (2.8)	Reference			
Homemaker	1.4 (2.8)	-0.58 (-1.17, 0.02)	0.05	-0.02 (-0.45, 0.42)	0.93
Co-morbidity					
No	1.6 (2.7)	Reference			
Yes	1.4 (3.0)	-0.20 (-0.73, 0.34)	0.48		
Past history of hormonal therapy					
No	1.5 (2.8)	Reference			
Yes	1.3 (3.1)	-0.22 (-2.02, 1.57)	0.80		
Past history of Fractures					
No	1.5 (2.7)	Reference			
Yes	1.0 (3.5)	-0.60 (-1.54, 0.34)	0.21		
Age at menarche	1.5 (2.8)	0.18 (-0.01, 0.36)	0.05	0.02 (-0.11, 0.15)	0.77
Status of Menopause					
No	2.9 (2.1)	Reference			
Yes	0.6 (2.8)	-2.37 (-2.88, -1.86)	<0.001	-0.02 (-0.52, 0.48)	0.95
Waist Hip Ratio (WHR)	1.5 (2.8)	-6.16 (-9.9, -2.3)	0.001	-5.78 (-8.55, -3.01)	<0.001
Years since menopause	1.5 (2.8)	-0.2 (-0.2, -0.1)	<0.001	-	

## Discussion

This community-based cross-sectional study conducted to estimate osteoporosis risk among postmenopausal women revealed that approximately 20% of participants were at risk of osteoporosis, with a notable increase in risk observed among those aged 55 years and above. A study conducted by Khinda et al. [15] in 2022 to estimate the prevalence of osteoporosis among postmenopausal women in Punjab found a prevalence of 30.5%. This difference in estimates could be attributed to several factors. Firstly, differences in age distribution may have contributed, as our study participants had a mean age of 54.5 years, whereas Khinda et al. reported a higher mean age of 68.5 years among women with osteoporosis. Aging is a known risk factor for osteoporosis, with bone density typically declining with age [16], which could explain the higher prevalence observed in the older population studied by Khinda et al.

Furthermore, variations in body mass index (BMI) between the study populations may have influenced prevalence estimates. Our study participants had a lower mean BMI (17.2) compared to the study by Khinda et al. (23.5) among women with osteoporosis. Studies have shown that BMI is directly associated with osteoporosis risk, with higher BMI being a risk factor for the condition [17]. Moreover, the differences in methodology may have played a role in the variation in prevalence estimates.

Years since menopause (YSM) serves as a crucial determinant of osteoporosis risk, with a shorter duration since menopause indicating a potentially lower risk. In our study, the mean YSM was 10.2 years, which was relatively shorter compared to the study conducted by Khinda et al., where the mean YSM was 12.8 years. This discrepancy in YSM between the two studies could contribute to the variation in osteoporosis prevalence estimates, as a longer duration since menopause is associated with a higher risk of osteoporosis development. In our study, we utilized a community-based screening tool, OSTA, to identify postmenopausal women at risk of osteoporosis. In contrast, Khinda et al. employed DEXA, considered the gold standard for diagnosing osteoporosis. The choice of diagnostic tool may have influenced prevalence estimates, as DEXA provides a more comprehensive assessment of bone mineral density compared to OSTA.

Another study using similar methodology was conducted by Muslim et al. [10] in Malay women in 2012. They found the prevalence of osteoporosis as 46% which is much higher than our study’s estimate. This disparity in prevalence estimates could be due to the demographic characteristics of the study populations, with the latter encompassed an older cohort, as indicated by the higher mean age of participants (59.4 years) compared to our study. Age is a well-established risk factor for osteoporosis, with bone density typically declining with advancing age, thus potentially contributing to the higher prevalence observed in their study. Moreover, differences in body composition may have played a role in the contrasting prevalence estimates. Specifically, the mean BMI of women with osteoporosis was substantially higher in the Muslim et al. study (26.3) than in ours (17.2). Additionally, variations in the timing of menarche, the onset of menstruation, between the study populations may have contributed to differences in osteoporosis prevalence. The mean age of menarche was lower in the study by Muslim et al. (13.4 years) compared to ours (14.2 years). Early menarche has been associated with increased peak bone mass and potentially lower risk of osteoporosis later in life [18]. Therefore, the earlier onset of menstruation in their study cohort may have conferred a protective effect against osteoporosis, influencing the prevalence estimates.

Race and ethnicity are known to display considerable influence on osteoporosis development due to variations in bone density and fracture risk among different population groups. These differences are often attributed to inherent disparities in bone metabolism and body composition. Evidence suggests that individuals of Asian descent tend to have lower bone mineral density compared to Caucasians, placing them at increased risk for osteoporosis and related fractures. Moreover, factors such as dietary habits, physical activity levels, and genetic predispositions vary among racial and ethnic groups, further influencing b and one health outcomes. Therefore, acknowledging the impact of race and ethnicity on osteoporosis risk is essential for developing targeted prevention and treatment strategies tailored to specific population groups [19].

Another study using the OSTA tool was conducted by Wang et al. [11] among women in Singapore in 2019 and found the prevalence of risk of osteoporosis as 64%. The important difference lies in the age distribution of participants. Wang et al. focused on women aged above 60 years, indicating an older population compared to ours. Advanced age is a well-established risk factor for osteoporosis due to age-related declines in bone density and increased prevalence of comorbidities. Older women also tend to have a higher likelihood of medication use for chronic conditions, which may further exacerbate bone loss and elevate the risk of osteoporosis. These age-related factors, combined with the higher prevalence of comorbidities in older populations, likely contributed to the elevated prevalence of osteoporosis. All the factors might contribute to the higher prevalence of risk of osteoporosis.

In our study, we found age to be an important determinant of developing osteoporosis. For each one-unit increase in age, the OSTA score decreases by an average of 0.20 (adjusted for other variables), indicating that older individuals tend to have lower OSTA scores, which implies a higher risk of osteoporosis. This resonates with the findings from other studies [11,15]. Furthermore, higher educational attainment, as reflected by schooling and diploma qualifications, was linked to higher OSTA scores in our study, indicating a lower risk of osteoporosis among individuals with more education. This finding is corroborated by a study conducted by Wang et al., emphasizing the potential protective effect of education against osteoporosis development. However, the association between occupation and OSTA scores was not statistically significant in our study, with homemakers exhibiting slightly lower OSTA scores compared to employed individuals. While this contrasts with findings from other studies that have reported a significant association between occupation and osteoporosis risk [11,15]. These findings underscore the complex interplay of socio-demographic factors in influencing osteoporosis risk and highlight the need for further research to elucidate the underlying mechanisms driving these associations.

In our study, we identified a statistically significant association between WHR and osteoporosis risk, aligning with findings from similar studies conducted in India. Interestingly, while previous research has often highlighted the significance of BMI in relation to osteoporosis risk, our study focused on WHR as a potential indicator. This underscores the importance of considering different anthropometric measures when assessing osteoporosis risk, as they may capture distinct aspects of body composition that influence bone health. Despite the well-established links between osteoporosis and factors such as comorbidities, hormonal therapy, and fracture history, we did not observe statistically significant associations with these variables in our study. One plausible explanation for this finding could be the sample size limitation, as our study was primarily designed to determine the prevalence of osteoporosis risk rather than to evaluate associations with specific risk factors. Therefore, future research with larger sample sizes may be warranted to explore these relationships more comprehensively.

Additionally, our study revealed an association between parity and osteoporosis risk, with women having more than two children exhibiting lower OSTA scores compared to those with fewer children. However, this association was not statistically significant after adjusting for other variables. While parity has been inconsistently linked to osteoporosis risk in previous studies, the mechanisms underlying this association remain unclear and warrant further investigation [20]. It is possible that factors such as hormonal fluctuations during pregnancy and lactation may influence bone density, but additional research is needed to elucidate the complex interplay between parity and osteoporosis risk. Therefore, it is essential to thoroughly assess and evaluate postmenopausal women to determine their susceptibility to osteoporosis, taking into account the above-mentioned risk factors.

Role of a primary care physician

The role of primary care physicians is pivotal in both identifying and managing osteoporosis, particularly in rural communities where access to specialized care may be limited. Evidence suggests that the knowledge, confidence and awareness about managing osteoporosis was low and considered osteoporosis as a low priority [21]. Another survey among the primary care physicians revealed insufficient knowledge and low level of osteoporosis screening in the primary care setting [22]. Through comprehensive training, primary care physicians can enhance their ability to recognize osteoporosis risk factors and management skills during their routine health assessments and screenings. This includes evaluating medical history, lifestyle factors, and performing physical examinations to identify individuals at risk. Additionally, primary care physicians can utilize validated tools such as the OSTA scale to assess osteoporosis risk and guide further diagnostic evaluations. Early identification allows for targeted interventions, such as lifestyle modifications (e.g., diet, exercise), optimizing calcium and vitamin D intake, and initiating pharmacological treatment when indicated. Moreover, primary care physicians play a crucial role in patient education, emphasizing the importance of fall prevention strategies and adherence to prescribed treatments to mitigate the risk of fractures. By integrating osteoporosis management into primary care practices and providing tailored interventions, primary care physicians can effectively reduce the burden of osteoporosis-related complications in rural communities, promoting better long-term outcomes and quality of life for their patients [23].

Strength and limitations

This study possesses several strengths. To the best of our knowledge, this study is the first community-based study within a rural population in India to assess osteoporosis risk utilizing the OSTA scale. The high response rate of 98.8% ensured minimization of selection bias and enhanced the credibility of the findings. With a sample size of 435 postmenopausal women, the study had adequate statistical power to estimate osteoporosis risk accurately. Methodologically, rigorous measures were employed, including the calibration of instruments for measuring anthropometric measurements, comprehensive training provided to data collectors, and adherence to standardized protocols. These steps ensure the reliability and accuracy of the collected data and osteoporosis risk assessment was done using the validated OSTA with high sensitivity. Additionally, multivariable regression analysis was conducted to explore associations between various factors and osteoporosis risk, allowing for a nuanced understanding of determinants. However, limitations certain exist, including the study design’s inability to establish causality between risk factors and osteoporosis. Reliance on self-reported data may introduce recall bias, particularly for medical history and reproductive factors. The study was conducted in one village, a homogeneous rural population in Chengalpattu district may limit generalizability to other populations. Despite adjusting for covariates, residual confounding from unmeasured factors may persist, impacting the validity of the results.

## Conclusions

This community-based study reveals osteoporosis risk among postmenopausal women as 20% using the OSTA scale, highlighting age and waist-hip ratio as a key determinant. Our findings underscore the critical need for early identification and intervention strategies, particularly targeting older and obese individuals, to alleviate the burden of osteoporosis and associated fractures in rural communities. Community-level initiatives such as health education programs, promotion of regular physical activity to decrease body fat, and ensuring access to fortified foods rich in calcium and vitamin D are vital for fostering preventive behaviours. Advocating for policies that support healthy aging, including adequate nutrition, physical activity, and healthcare access, is essential for addressing the growing osteoporosis burden in rural populations. Furthermore, longitudinal studies encompassing diverse populations are warranted to further elucidate osteoporosis epidemiology and inform evidence-based policies and interventions aimed at mitigating its public health impact.
